# 5,7,13,15-Tetra­oxo-2,2,10,10-tetra­kis­(trifluoro­meth­yl)-4,8,12,16-tetra­oxa-1(1,4),3(1,4),6(1,2),9(1,4),11(1,4),14(1,2)-hexa­benzenahexa­deca­phane tetra­hydro­furan monosolvate

**DOI:** 10.1107/S1600536812011166

**Published:** 2012-03-21

**Authors:** Qing-Zhong Guo, Yi Du

**Affiliations:** a School of Materials Science and Engineering, Wuhan Institute of Technology, Wuhan 430073, People’s Republic of China

## Abstract

The title compound, C_46_H_24_F_12_O_8_·C_4_H_8_O, consists of a cyclic aryl ester dimer and a tetra­hydro­furan mol­ecule. In the structure of the cyclic dimer, one carbonyl group stretches above the cavity and the other below.

## Related literature

For related structures of the cyclic aryl ester dimer, *cyclo*-bis­[1,4-phenyl­ene(hexa­fluoro­isopropyl­idene)phthalate] tetra­hydro­furan monosolvent, see: Jiang *et al.* (1997*b* ▶); Teasley *et al.* (1998 ▶); Qi *et al.* (1999 ▶); Guo *et al.* (2003 ▶). For the use of ring-opening polymerization (ROP) reactions of cyclic aryl oligomers in the preparation of high performance aromatic polymers, see: Brunelle (2008 ▶); Brunelle *et al.* (1990 ▶); Chan *et al.* (1995 ▶); Jiang *et al.* (1997*a* ▶). For ideal bond angles, see: Coulter & Windle (1989 ▶);
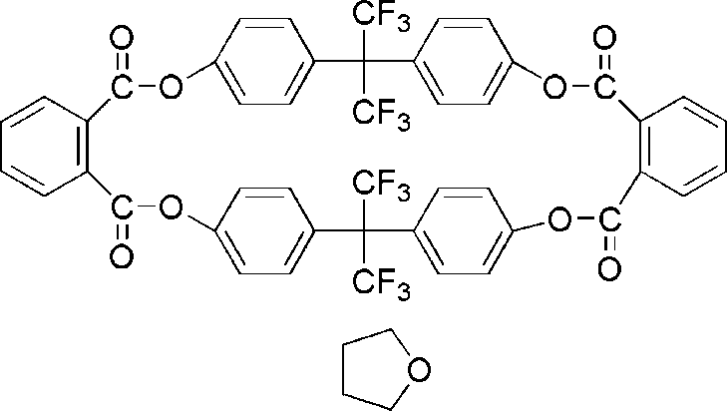


## Experimental

### 

#### Crystal data

C_46_H_24_F_12_O_8_·C_4_H_8_O
*M* *_r_* = 1004.76
Triclinic, 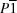

*a* = 9.3857 (17) Å
*b* = 11.2748 (17) Å
*c* = 12.615 (2) Å
α = 105.715 (14)°
β = 97.969 (14)°
γ = 103.167 (14)°
*V* = 1222.4 (3) Å^3^
*Z* = 1
Mo *K*α radiation
μ = 0.12 mm^−1^
*T* = 293 K
0.43 × 0.33 × 0.30 mm

#### Data collection

Siemens P4 diffractometer
Absorption correction: ψ scan (*XSCANS*; Bruker, 2001 ▶) *T*_min_ = 0.950, *T*_max_ = 0.964
5660 measured reflections
4684 independent reflections
1916 reflections with *I* > 2σ(*I*)
*R*_int_ = 0.022
3 standard reflections every 197 reflections intensity decay: 2.2%

#### Refinement

*R*[*F* ^2^ > 2σ(*F* ^2^)] = 0.065
*wR*(*F* ^2^) = 0.158
*S* = 1.00
4684 reflections
344 parameters
H-atom parameters constrained
Δρ_max_ = 0.32 e Å^−3^
Δρ_min_ = −0.20 e Å^−3^

### 

Data collection: *XSCANS* (Bruker, 2001 ▶); cell refinement: *XSCANS*; data reduction: *XSCANS*; program(s) used to solve structure: *SHELXS97* (Sheldrick, 2008 ▶); program(s) used to refine structure: *SHELXL97* (Sheldrick, 2008 ▶); molecular graphics: *SHELXTL* (Sheldrick, 2008 ▶); software used to prepare material for publication: *SHELXTL*.

## Supplementary Material

Crystal structure: contains datablock(s) I, global. DOI: 10.1107/S1600536812011166/zj2060sup1.cif

Structure factors: contains datablock(s) I. DOI: 10.1107/S1600536812011166/zj2060Isup2.hkl

Additional supplementary materials:  crystallographic information; 3D view; checkCIF report

## Figures and Tables

**Fig. 1.**
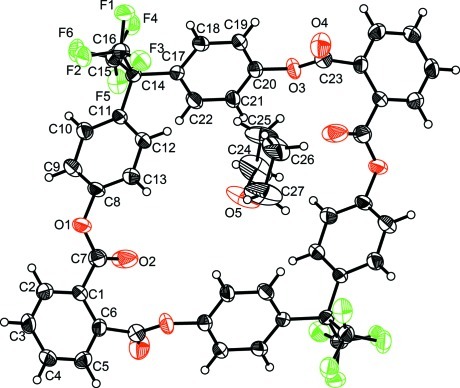
[Crystal structure of the title compound with ellipsoids of non-hydrogen atoms drawn at the 30% probability level.]

**Fig. 2.**
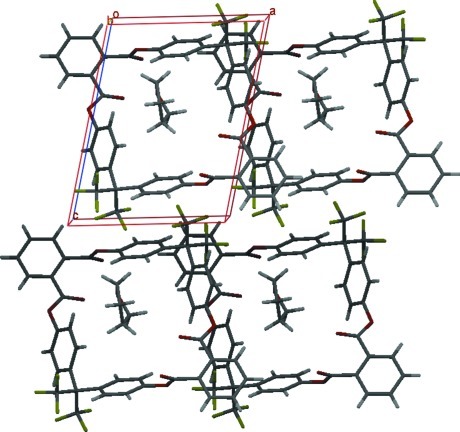
[The packing structure of the title complex. The C—O and C—F bonds are shown as red and yellowish-green thick bond mode for clarity.]

## supplementary crystallographic information

### Comment

Ring-opening polymerization (ROP) reactions constitute an important class of polymerization reactions. The advantages of using ROP of cyclic aryl oligomers to prepare high performance aromatic thermoplastics, such as polycarbonate and poly(aryl ester)s, have been widely recognized in recent years (Brunelle *et al.*, 1990; Brunelle, 2008; Chan *et al.*, 1995; Jiang *et al.* 1997*a*). It is generally believed that the ROP of aromatic cyclic oligomers is essentially thermoneutral and driven by entropy changes as the cyclic oligomers have big size with little or no ring strain. In order to obtain decisive evidence of the macrocyclic structure and investigate the nature of ROP, the single-crystal X-ray structure of cyclic ester dimer, the title compound, was determined.

The structure of cyclic dimer, *cyclo*-Bis[1,4-phenylene(hexafluoroisopropylidene)-phthalate (shown in Fig. 1) exhibits two types of conformation about ester groups. One of the carbonyl groups stretch above the cavity of the cyclic structure and the others stretch beneath the cavity. The interplanar dihedral angle of the phenyls attached to the hexafluoroisopropylidene is 69.67°. The distance between C(14) and its symmetrical carbon atom is 1.0729 nm. The bond angles at C7—O1—C8 of 119.6° and O3—C23(O4)—C6^i^ of 111.0° are all close to the idealized values of 118. 8° and 111.7°, respectively (Jiang *et al.*, 1997*b*; Coulter & Windle, 1989). The phenyl rings in cyclic dimer have a good planarity (root mean square deviations from the planarity of the phenyl planes are 0.00043, 0.00069 and 0.00053 nm, respectively). Overall, X-ray analysis indicates that the cyclic dimer is constructed without severe internal strain. This result indicates that the ROP of cyclic aryl ester dimer is driven by entropy changes and provides a base for the study on the mechanism of ROP reaction and the relationship between the cyclic nature and ROP reaction.

### Experimental

The cyclization reaction was conducted in a 500 ml threeneck round-bottom flask charged with 150 ml dichloromethane, 30 ml distilled water and 0.16 g cetyltrimethylammonium bromide at room temperature. A solution of phthaloyl dichloride (1.014 g, 5 mmol) in 50 ml dichloromethane and a solution of disodium salt of 4,4'-(hexafluoroisopropylidene) diphenol (1.682 g, 5 mmol) in 50 ml distilled water were delivered into the mechanically stirred flask in an equimolar fashion over an 8 h period. After the addition, the mixture was stirred for another 2 h to ensure complete reaction. The organic phase was separated by a separating funnel and extracted with distilled water three times and then evaporated to dryness. The colorless cyclic dimer was obtained by recrystallization from tetrahydrofuran (THF). The isolated yield of cyclic dimer was 1.3 g (54.7% yield). Colorless block crystals suitable for X-ray analysis were obtained by slow evaporation from a THF solution at room temperature for about one week.

### Refinement

The H atoms were placed in idealized positions and allowed to ride on the relevant carbon atoms, with C—H = 0.93Å and *Uiso*(H) = 1.0*Ueq*(C) except for in THF, where C—H = 0.97 Å.

### Crystal data

**Table:**

C_46_H_24_F_12_O_8_·C_4_H_8_O	*Z* = 1
*M**_r_* = 1004.76	*F*(000) = 512
Triclinic, *P*1	*D*_x_ = 1.365 Mg m^−^^3^
*a* = 9.3857 (17) Å	Mo *K*α radiation, λ = 0.71073 Å
*b* = 11.2748 (17) Å	Cell parameters from 23 reflections
*c* = 12.615 (2) Å	θ = 9.5–20.1°
α = 105.715 (14)°	µ = 0.12 mm^−^^1^
β = 97.969 (14)°	*T* = 293 K
γ = 103.167 (14)°	Block, colorless
*V* = 1222.4 (3) Å^3^	0.43 × 0.33 × 0.30 mm

### Data collection

**Table:**

Siemens P4 diffractometer	1916 reflections with *I* > 2σ(*I*)
Radiation source: fine-focus sealed tube	*R*_int_ = 0.022
Graphite monochromator	θ_max_ = 26.0°, θ_min_ = 4.0°
ω scans	*h* = −1→11
Absorption correction: ψ scan (*XSCANS*; Bruker, 2001)	*k* = −13→13
*T*_min_ = 0.950, *T*_max_ = 0.964	*l* = −15→15
5660 measured reflections	3 standard reflections every 197 reflections
4684 independent reflections	intensity decay: 2.2%

### Refinement

**Table:**

Refinement on *F*^2^	Primary atom site location: structure-invariant direct methods
Least-squares matrix: full	Secondary atom site location: difference Fourier map
*R*[*F*^2^ > 2σ(*F*^2^)] = 0.065	Hydrogen site location: inferred from neighbouring sites
*wR*(*F*^2^) = 0.158	H-atom parameters constrained
*S* = 1.00	*w* = 1/[σ^2^(*F*_o_^2^) + (0.052*P*)^2^] where *P* = (*F*_o_^2^ + 2*F*_c_^2^)/3
4684 reflections	(Δ/σ)_max_ = 0.001
344 parameters	Δρ_max_ = 0.32 e Å^−^^3^
0 restraints	Δρ_min_ = −0.20 e Å^−^^3^

### Special details

**Table:**

Geometry. All e.s.d.'s (except the e.s.d. in the dihedral angle between two l.s. planes) are estimated using the full covariance matrix. The cell e.s.d.'s are taken into account individually in the estimation of e.s.d.'s in distances, angles and torsion angles; correlations between e.s.d.'s in cell parameters are only used when they are defined by crystal symmetry. An approximate (isotropic) treatment of cell e.s.d.'s is used for estimating e.s.d.'s involving l.s. planes.
Refinement. Refinement of *F*^2^ against ALL reflections. The weighted *R*-factor *wR* and goodness of fit *S* are based on *F*^2^, conventional *R*-factors *R* are based on *F*, with *F* set to zero for negative *F*^2^. The threshold expression of *F*^2^ > σ(*F*^2^) is used only for calculating *R*-factors(gt) *etc*. and is not relevant to the choice of reflections for refinement. *R*-factors based on *F*^2^ are statistically about twice as large as those based on *F*, and *R*- factors based on ALL data will be even larger.

### Fractional atomic coordinates and isotropic or equivalent isotropic displacement parameters (Å^2^)

**Table:**

	*x*	*y*	*z*	*U*_iso_*/*U*_eq_	Occ. (<1)
O1	−0.0470 (3)	−0.3086 (2)	−0.5363 (2)	0.0794 (8)	
O2	0.1569 (4)	−0.3494 (4)	−0.5872 (3)	0.1347 (15)	
O3	0.8188 (3)	0.3985 (2)	−0.1740 (2)	0.0715 (8)	
O4	0.7764 (4)	0.5631 (3)	−0.2264 (3)	0.1049 (11)	
F1	0.1508 (2)	0.2999 (2)	−0.0496 (2)	0.1018 (9)	
F2	−0.0143 (2)	0.1254 (2)	−0.1459 (2)	0.0908 (8)	
F3	0.1038 (3)	0.2448 (2)	−0.2304 (2)	0.0860 (7)	
F4	0.3519 (3)	0.2028 (2)	0.04653 (17)	0.1021 (9)	
F5	0.3492 (3)	0.0118 (3)	−0.0450 (2)	0.0933 (8)	
F6	0.1484 (3)	0.0505 (2)	−0.00260 (18)	0.0977 (8)	
C1	−0.0778 (4)	−0.4798 (3)	−0.7009 (3)	0.0589 (10)	
C2	−0.2286 (4)	−0.5213 (4)	−0.7051 (3)	0.0792 (12)	
H2	−0.2674	−0.4835	−0.6448	0.079*	
C3	−0.3231 (4)	−0.6168 (4)	−0.7958 (3)	0.0827 (13)	
H3	−0.4245	−0.6449	−0.7968	0.083*	
C4	−0.2656 (5)	−0.6701 (4)	−0.8850 (3)	0.0721 (11)	
H4	−0.3286	−0.7347	−0.9475	0.072*	
C5	−0.1159 (5)	−0.6294 (4)	−0.8830 (3)	0.0706 (11)	
H5	−0.0781	−0.6659	−0.9444	0.071*	
C6	−0.0203 (4)	−0.5338 (3)	−0.7899 (3)	0.0572 (9)	
C7	0.0240 (5)	−0.3755 (4)	−0.6024 (3)	0.0736 (11)	
C8	0.0367 (4)	−0.2045 (3)	−0.4396 (3)	0.0614 (10)	
C9	0.0224 (4)	−0.2157 (3)	−0.3377 (4)	0.0724 (11)	
H9	−0.0326	−0.2923	−0.3313	0.072*	
C10	0.0913 (4)	−0.1106 (3)	−0.2421 (3)	0.0658 (10)	
H10	0.0832	−0.1175	−0.1712	0.066*	
C11	0.1709 (3)	0.0029 (3)	−0.2513 (3)	0.0498 (8)	
C12	0.1826 (4)	0.0095 (3)	−0.3588 (3)	0.0617 (10)	
H12	0.2381	0.0852	−0.3665	0.062*	
C13	0.1140 (4)	−0.0932 (4)	−0.4529 (3)	0.0680 (10)	
H13	0.1198	−0.0874	−0.5244	0.068*	
C14	0.2397 (3)	0.1268 (3)	−0.1490 (3)	0.0520 (9)	
C15	0.1194 (4)	0.2006 (4)	−0.1444 (4)	0.0738 (12)	
C16	0.2704 (5)	0.0965 (5)	−0.0377 (3)	0.0737 (12)	
C17	0.3893 (4)	0.2048 (3)	−0.1606 (3)	0.0495 (8)	
C18	0.4309 (4)	0.3383 (3)	−0.1327 (3)	0.0635 (10)	
H18	0.3630	0.3834	−0.1104	0.063*	
C19	0.5725 (4)	0.4043 (3)	−0.1380 (3)	0.0640 (10)	
H19	0.5997	0.4934	−0.1176	0.064*	
C20	0.6714 (4)	0.3391 (3)	−0.1729 (3)	0.0545 (9)	
C21	0.6330 (4)	0.2069 (3)	−0.2027 (3)	0.0603 (10)	
H21	0.7010	0.1625	−0.2264	0.060*	
C22	0.4938 (4)	0.1420 (3)	−0.1968 (3)	0.0633 (10)	
H22	0.4681	0.0529	−0.2178	0.063*	
C23	0.8577 (5)	0.5007 (4)	−0.2080 (3)	0.0692 (11)	
O5	0.6163 (12)	−0.0885 (7)	−0.3822 (10)	0.172 (4)	0.50
C24	0.5149 (18)	−0.1575 (12)	−0.4794 (11)	0.152 (5)	0.50
H24A	0.5250	−0.1136	−0.5355	0.152*	0.50
H24B	0.4140	−0.1683	−0.4663	0.152*	0.50
C25	0.5431 (12)	−0.2781 (11)	−0.5174 (9)	0.123 (4)	0.50
H25A	0.4539	−0.3425	−0.5655	0.123*	0.50
H25B	0.6229	−0.2735	−0.5588	0.123*	0.50
C26	0.5847 (17)	−0.3060 (8)	−0.4206 (11)	0.140 (5)	0.50
H26A	0.6684	−0.3428	−0.4242	0.140*	0.50
H26B	0.5020	−0.3652	−0.4070	0.140*	0.50
C27	0.632 (2)	−0.1677 (12)	−0.3238 (8)	0.177 (7)	0.50
H27A	0.5667	−0.1657	−0.2706	0.177*	0.50
H27B	0.7349	−0.1466	−0.2832	0.177*	0.50

### Atomic displacement parameters (Å^2^)

**Table:**

	*U*^11^	*U*^22^	*U*^33^	*U*^12^	*U*^13^	*U*^23^
O1	0.0608 (16)	0.0640 (16)	0.0805 (17)	0.0018 (14)	0.0152 (15)	−0.0173 (14)
O2	0.065 (2)	0.153 (3)	0.113 (3)	0.010 (2)	0.0058 (19)	−0.052 (2)
O3	0.0525 (16)	0.0579 (16)	0.100 (2)	−0.0004 (13)	0.0185 (14)	0.0299 (15)
O4	0.094 (2)	0.104 (2)	0.148 (3)	0.045 (2)	0.048 (2)	0.064 (2)
F1	0.0708 (15)	0.0862 (16)	0.1111 (19)	0.0068 (13)	0.0357 (14)	−0.0247 (14)
F2	0.0465 (13)	0.0826 (15)	0.126 (2)	0.0043 (12)	0.0302 (13)	0.0097 (14)
F3	0.0726 (15)	0.0738 (15)	0.1102 (19)	0.0284 (12)	0.0150 (14)	0.0219 (14)
F4	0.0846 (17)	0.124 (2)	0.0530 (13)	−0.0261 (15)	0.0017 (12)	0.0078 (13)
F5	0.0768 (17)	0.115 (2)	0.0878 (17)	0.0116 (15)	0.0018 (13)	0.0510 (16)
F6	0.0713 (15)	0.125 (2)	0.0685 (14)	−0.0213 (14)	0.0194 (12)	0.0227 (14)
C1	0.053 (2)	0.048 (2)	0.058 (2)	−0.0016 (18)	0.0121 (18)	0.0029 (17)
C2	0.058 (3)	0.080 (3)	0.073 (3)	−0.002 (2)	0.023 (2)	−0.006 (2)
C3	0.058 (2)	0.086 (3)	0.075 (3)	−0.020 (2)	0.016 (2)	0.010 (2)
C4	0.069 (3)	0.063 (2)	0.057 (2)	−0.007 (2)	0.006 (2)	0.0006 (19)
C5	0.071 (3)	0.084 (3)	0.047 (2)	0.009 (2)	0.018 (2)	0.012 (2)
C6	0.048 (2)	0.062 (2)	0.056 (2)	0.0037 (18)	0.0144 (18)	0.0183 (19)
C7	0.046 (2)	0.081 (3)	0.071 (3)	0.005 (2)	0.005 (2)	0.000 (2)
C8	0.050 (2)	0.050 (2)	0.061 (2)	0.0018 (18)	0.0037 (19)	−0.0053 (19)
C9	0.076 (3)	0.048 (2)	0.078 (3)	0.003 (2)	0.008 (2)	0.011 (2)
C10	0.074 (3)	0.060 (2)	0.054 (2)	0.001 (2)	0.0122 (19)	0.0171 (19)
C11	0.0417 (19)	0.0453 (19)	0.051 (2)	0.0035 (16)	0.0076 (16)	0.0046 (16)
C12	0.066 (2)	0.049 (2)	0.056 (2)	−0.0016 (18)	0.0127 (19)	0.0071 (18)
C13	0.074 (3)	0.063 (2)	0.052 (2)	0.002 (2)	0.0131 (19)	0.0086 (19)
C14	0.0427 (19)	0.058 (2)	0.046 (2)	0.0087 (17)	0.0096 (16)	0.0051 (16)
C15	0.055 (3)	0.068 (3)	0.075 (3)	0.006 (2)	0.020 (2)	−0.009 (2)
C16	0.057 (3)	0.088 (3)	0.056 (3)	−0.003 (2)	0.011 (2)	0.010 (2)
C17	0.044 (2)	0.048 (2)	0.050 (2)	0.0071 (16)	0.0143 (16)	0.0064 (16)
C18	0.055 (2)	0.052 (2)	0.071 (2)	0.0101 (19)	0.0199 (19)	0.0003 (18)
C19	0.058 (2)	0.046 (2)	0.077 (2)	0.0033 (19)	0.023 (2)	0.0071 (18)
C20	0.042 (2)	0.054 (2)	0.060 (2)	0.0075 (18)	0.0108 (17)	0.0113 (18)
C21	0.048 (2)	0.048 (2)	0.084 (3)	0.0100 (18)	0.0246 (19)	0.0163 (19)
C22	0.057 (2)	0.0437 (19)	0.078 (3)	0.0058 (18)	0.012 (2)	0.0091 (18)
C23	0.071 (3)	0.062 (3)	0.070 (3)	0.012 (2)	0.020 (2)	0.017 (2)
O5	0.195 (9)	0.071 (5)	0.184 (9)	0.023 (5)	−0.018 (8)	−0.026 (6)
C24	0.203 (14)	0.113 (10)	0.132 (10)	0.048 (10)	−0.041 (10)	0.062 (9)
C25	0.114 (8)	0.112 (8)	0.094 (8)	0.058 (7)	−0.053 (6)	−0.033 (7)
C26	0.216 (14)	0.052 (6)	0.168 (12)	0.046 (7)	0.043 (11)	0.050 (7)
C27	0.33 (2)	0.123 (10)	0.048 (5)	0.059 (12)	−0.041 (9)	0.020 (6)

### Geometric parameters (Å, º)

**Table:**

O1—C7	1.324 (4)	C11—C14	1.554 (4)
O1—C8	1.421 (4)	C12—C13	1.368 (5)
O2—C7	1.190 (4)	C12—H12	0.9300
O3—C23	1.328 (4)	C13—H13	0.9300
O3—C20	1.397 (4)	C14—C17	1.526 (4)
O4—C23	1.189 (4)	C14—C16	1.538 (5)
F1—C15	1.341 (4)	C14—C15	1.547 (5)
F2—C15	1.339 (4)	C17—C22	1.393 (5)
F3—C15	1.314 (5)	C17—C18	1.396 (4)
F4—C16	1.348 (4)	C18—C19	1.387 (5)
F5—C16	1.326 (5)	C18—H18	0.9300
F6—C16	1.331 (4)	C19—C20	1.359 (5)
C1—C6	1.367 (5)	C19—H19	0.9300
C1—C2	1.375 (5)	C20—C21	1.381 (4)
C1—C7	1.486 (5)	C21—C22	1.366 (5)
C2—C3	1.368 (5)	C21—H21	0.9300
C2—H2	0.9300	C22—H22	0.9300
C3—C4	1.367 (5)	C23—C6^i^	1.493 (5)
C3—H3	0.9300	O5—C27	1.323 (12)
C4—C5	1.370 (5)	O5—C24	1.355 (12)
C4—H4	0.9300	C24—C25	1.413 (14)
C5—C6	1.388 (5)	C24—H24A	0.9700
C5—H5	0.9300	C24—H24B	0.9700
C6—C23^i^	1.493 (5)	C25—C26	1.369 (13)
C8—C9	1.348 (5)	C25—H25A	0.9700
C8—C13	1.364 (5)	C25—H25B	0.9700
C9—C10	1.391 (5)	C26—C27	1.619 (14)
C9—H9	0.9300	C26—H26A	0.9700
C10—C11	1.370 (4)	C26—H26B	0.9700
C10—H10	0.9300	C27—H27A	0.9700
C11—C12	1.396 (5)	C27—H27B	0.9700
			
C7—O1—C8	119.6 (3)	F5—C16—F6	106.9 (4)
C23—O3—C20	123.7 (3)	F5—C16—F4	106.5 (4)
C6—C1—C2	119.5 (3)	F6—C16—F4	106.1 (3)
C6—C1—C7	119.1 (3)	F5—C16—C14	111.4 (3)
C2—C1—C7	121.4 (4)	F6—C16—C14	114.8 (3)
C3—C2—C1	121.6 (4)	F4—C16—C14	110.8 (4)
C3—C2—H2	119.2	C22—C17—C18	117.1 (3)
C1—C2—H2	119.2	C22—C17—C14	119.3 (3)
C4—C3—C2	118.8 (4)	C18—C17—C14	123.5 (3)
C4—C3—H3	120.6	C19—C18—C17	120.7 (3)
C2—C3—H3	120.6	C19—C18—H18	119.6
C3—C4—C5	120.5 (4)	C17—C18—H18	119.6
C3—C4—H4	119.7	C20—C19—C18	120.1 (3)
C5—C4—H4	119.7	C20—C19—H19	120.0
C4—C5—C6	120.3 (4)	C18—C19—H19	120.0
C4—C5—H5	119.8	C19—C20—C21	120.6 (3)
C6—C5—H5	119.8	C19—C20—O3	123.6 (3)
C1—C6—C5	119.2 (3)	C21—C20—O3	115.6 (3)
C1—C6—C23^i^	124.0 (3)	C22—C21—C20	119.2 (3)
C5—C6—C23^i^	116.8 (3)	C22—C21—H21	120.4
O2—C7—O1	122.7 (4)	C20—C21—H21	120.4
O2—C7—C1	123.6 (4)	C21—C22—C17	122.2 (3)
O1—C7—C1	113.6 (3)	C21—C22—H22	118.9
C9—C8—C13	122.5 (3)	C17—C22—H22	118.9
C9—C8—O1	117.6 (3)	O4—C23—O3	124.5 (4)
C13—C8—O1	119.5 (4)	O4—C23—C6^i^	124.3 (4)
C8—C9—C10	118.8 (4)	O3—C23—C6^i^	111.0 (4)
C8—C9—H9	120.6	C27—O5—C24	107.4 (9)
C10—C9—H9	120.6	O5—C24—C25	107.4 (9)
C11—C10—C9	120.7 (3)	O5—C24—H24A	110.2
C11—C10—H10	119.6	C25—C24—H24A	110.2
C9—C10—H10	119.6	O5—C24—H24B	110.2
C10—C11—C12	118.2 (3)	C25—C24—H24B	110.2
C10—C11—C14	123.3 (3)	H24A—C24—H24B	108.5
C12—C11—C14	118.4 (3)	C26—C25—C24	104.2 (9)
C13—C12—C11	121.2 (3)	C26—C25—H25A	110.9
C13—C12—H12	119.4	C24—C25—H25A	110.9
C11—C12—H12	119.4	C26—C25—H25B	110.9
C8—C13—C12	118.5 (4)	C24—C25—H25B	110.9
C8—C13—H13	120.7	H25A—C25—H25B	108.9
C12—C13—H13	120.7	C25—C26—C27	103.4 (7)
C17—C14—C16	106.5 (3)	C25—C26—H26A	111.1
C17—C14—C15	112.8 (3)	C27—C26—H26A	111.1
C16—C14—C15	108.8 (3)	C25—C26—H26B	111.1
C17—C14—C11	111.8 (3)	C27—C26—H26B	111.1
C16—C14—C11	111.9 (3)	H26A—C26—H26B	109.0
C15—C14—C11	105.2 (3)	O5—C27—C26	102.8 (7)
F3—C15—F2	107.9 (4)	O5—C27—H27A	111.2
F3—C15—F1	107.9 (4)	C26—C27—H27A	111.2
F2—C15—F1	105.3 (3)	O5—C27—H27B	111.2
F3—C15—C14	111.4 (3)	C26—C27—H27B	111.2
F2—C15—C14	111.4 (3)	H27A—C27—H27B	109.1
F1—C15—C14	112.6 (3)		

Symmetry code: (i) −*x*+1, −*y*, −*z*−1.
